# Protective Effect and Mechanism of Total Flavones from *Rhododendron simsii* Planch Flower on Cultured Rat Cardiomyocytes with Anoxia and Reoxygenation

**DOI:** 10.1155/2015/863531

**Published:** 2015-03-10

**Authors:** Yi Jiao, Yi-Fei Fan, Yu-Ling Wang, Jun-Yan Zhang, Shuo Chen, Zhi-Wu Chen

**Affiliations:** ^1^Department of Pharmacology, Anhui Medical University, Hefei, Anhui 230032, China; ^2^Xinglin College, Liaoning University of Traditional Chinese Medicine, Shenyang, Liaoning 110167, China

## Abstract

Many flavonoids have cardioprotection against myocardial ischemia/reperfusion (I/R) injury. Total flavones from *Rhododendron simsii* Planch flower (TFR) can protect myocardial ischemic injuries. However, its protective mechanism is still unknown. The present study was designed to investigate the mechanism of TFR on myocardial I/R and anoxia/reoxygenation (A/R) injuries. Rat model of myocardial I/R injury was made, and myocardial infarction was determined. A/R injury was induced in cultured rat cardiomyocytes; cellular damage was evaluated by measuring cell viability, LDH and cTnT releases, and MDA content. Expressions of ROCK_1_ and ROCK_2_ protein were examined by Western blot analysis, and K^+^ currents were recorded by using whole-cell patch clamp technique. TFR 20~80 mg/kg markedly reduced I/R-induced myocardial infarction. TFR 3.7~300 mg/L significantly inhibited A/R-induced reduction of cell viability, LDH and cTnT releases, and MDA production. Exposure to A/R significantly increased ROCK_1_ and ROCK_2_ expressions in rat cardiomyocytes, but TFR 33.3~300 mg/L obviously inhibited this increase. 300 mg/L TFR significantly augmented inward rectifier K^+^ current and other K^+^ currents in rat cardiomyocytes. These results indicate that TFR has a protective effect on rat cardiomyocytes A/R damage, and the protective mechanism may be engaged with the inhibition of ROCK_1_ and ROCK_2_ and activation of K^+^ channels.

## 1. Introduction

Ischemic cardiovascular disease, the most common heart disease, is the main cause of mortality and morbidity worldwide. Coronary arteries and their branches supply oxygen-rich blood to myocardium. Stopping of blood supply to the heart muscle leads to myocardial ischemic injury. Restoration of blood supply is essential to prevent irreversible injury. However, sudden blood flow returning to ischemic myocardium may paradoxically augment myocardial injury. This is referred to as myocardial ischemia/reperfusion (I/R) injury [1], characterized by myocardial inflammatory responses, metabolic disorder, cardiac dysfunction, and subsequent myocardial cell death. Alike, oxygen deprivation followed by reoxygenation causes anoxia and reoxygenation (A/R) injury.

The RhoA/Rho-kinase signaling pathway has an important role in many pathological processes. The Rho-kinase (Rho-associated coiled-coil forming protein kinase, ROCK) is a serine/threonine kinase belonging to the AGC (PKA/PKG/PKC) family. ROCK was identified as the first effectors of RhoA; it mediates some essential cellular functions including cell shape, motility, cellular contractility, proliferation, coronary vasospasm, and inflammation [2]. There are two isoforms of ROCK, ROCK_1_ and ROCK_2_. Recent studies demonstrate that ROCKs play a major role in the pathogenesis of myocardial infarction [3]. ROCK activation occurs in rat reperfused myocardium specifically, and this activation is deleterious. Moreover, inhibition of ROCKs has a protective effect against myocardial I/R injury in rat [4]. Y-27632, a ROCK inhibitor, significantly reduced I/R injury-induced infarct size and cardiomyocyte apoptosis by attenuating inflammatory responses [5]. These studies suggest that ROCK inhibition may be a novel therapeutic target for the treatment of ischemic cardiovascular diseases.

Multiple types of K^+^ channels are present in the cardiovascular system and play crucial roles. It is a known fact that K^+^ channel blocker can block the infarct-limiting effects of ischemic preconditioning and K^+^ channel openers mimic the protective effect [6]. The opening of ATP-sensitive K^+^ channel (K_ATP_ channel) could protect cardiac myocytes against ischemic injuries. The cardiac K_ATP_ channel consists of two distinct proteins, an inwardly rectifying potassium channel pore subunit (Kir6.2) and the sulfonylurea receptor (SUR2A). 5′-AMP-activated protein kinase (AMPK) is an energy sensor protein kinase that takes part in regulating cellular energy; it interacted with Kir6.2, subsequently triggers and promotes Kir6.2/K_ATP_ channel opening, and induces cardioprotection. However, Kir6.2 knockout could eliminate the cardioprotective effect of AMPK [4, 7]. Adenosine-mediated protection against I/R injury was found to be abolished by K_ATP_ channel inhibition in human myocardium [8]. Inward rectifier K^+^ channel is distinct from K_ATP_ channel, which plays an important role in the repolarization of cardiac myocytes. Inhibition of inward rectifier K^+^ channels (Kir2.1 or Kir2.2) abolished protection of ischemic preconditioning in rabbit cardiomyocytes [9].

Flavonoid compounds are widely distributed in many Chinese herbs and natural plants and have various biological activities and pharmacological functions including vasorelaxing as well as cardioprotection against myocardial I/R injury [10–12]. Therefore, Chinese herbs are important resources to develop valid and safe drugs for the treatment of diseases.* Rhododendron simsii* Planch flower, a Chinese herbal medicine, has been used for treating patients with bronchitis in China for thousands year. Total flavones from* Rhododendron simsii* Planch flower (TFR), an effective part extracted from* Rhododendron simsii* Planch flower, is comprised of flavones such as rutin, hyperin, quercetin, and other flavonoids [13, 14]. Our previous studies have shown that TFR has significant protective effects against myocardial or cerebral ischemic injuries in rabbit and rat [10–12]. However, its mechanism of cardioprotection remains poorly understood. Therefore, in the present study, the mechanism of TFR on myocardial I/R and A/R injury was investigated in rat model of I/R-induced myocardial infarction and cultured rat cardiomyocytes with A/R injury. By using the examination of ROCK protein expression, whole-cell patch clamp recording, and other approaches, the main focus in this study is on the roles of ROCK and K^+^ channels in the cardioprotection of TFR.

## 2. Material and Methods

### 2.1. Drugs and Reagents

TFR (content of flavones greater than 85%) was provided by Hefei Heyuan Medicine Technology Co., Ltd. (Hefei, China); nifedipine and BaCl_2_ were purchased from Sigma Co., USA; Y-27632 was purchased from Santa Cruz, USA; malondialdehyde (MDA) and lactate dehydrogenase (LDH) assay kits were purchased from Nanjing Jiancheng Biological Co., Nanjing, China; cardiac troponin T (cTnT) ELISA kits were provided by Shanghai Yuanye Biological Co., Shanghai, China; ROCK_1_ and ROCK_2_ were provided by Nanjing Enogene Biological Co., Nanjing, China.

### 2.2. Animals

Neonatal Sprague-Dawley rats (1~2 d old, half male and half female) and adult male Sprague-Dawley rats (weighing 300 to 350 g) were purchased from the Experimental Animal Center of Anhui Medical University. Rats were housed at 22 ± 2°C and relative humidity of 40 ± 5% under a 12-hour light/dark cycle. This investigation conforms to the regulations stipulated by Anhui Medical University animal care committee, which follows the protocol outlined in the Guide for the Care and Use of Laboratory Animals published by the US National Institutes of Health (NIH Publication no. 85-23, revised 1996).

### 2.3. Rat Myocardial I/R Injury Model

Rat myocardial I/R injury model was carried out according to previous method [10] with some modifications. Adult male Sprague-Dawley rat was anesthetized with 10% chloral hydrate (3 mL/kg) by peritoneal injection and placed in a supine position. Thoracotomy was performed by removing the left third rib to expose the heart, a 5-0 suture silk was placed around the left anterior descending coronary artery (LAD), which was 1-2 mm under the boundary of pulmonary conus and left auricle, and the ends of this ligature were passed through a small plastic tube to form a snare. The rat underwent 30 min of ischemia and then was released allowing reperfusion for a period of 90 min. At the end of reperfusion, LAD was ligated, and 0.25% Evans blue was administrated through femoral vein. Then the hearts were harvested and frozen at −20°C in a freezer. The heart was sectioned into five 2~3 mm transverse slices and incubated in 1% TTC in phosphate buffer (pH 7.4, 37°C) for 15 min. Infarct size (IS), area at risk (AAR), and left ventricle size of each slice were analyzed by ImageJ version 1.6 (National Institutes of Health, Bethesda, Md, USA).

The rats were randomly divided into the following 7 groups: sham group, I/R group, 1.6 mg/kg verapamil group, 30 mg/kg Y27632 group, 20 mg/kg TFR group, 40 mg/kg TFR group, and 80 mg/kg TFR group. Y27632 and verapamil were administrated by intravenous injection once a day for 5 days, and TFR was treated by gavage once a day for 5 days.

### 2.4. Primary Culture of Neonatal Rat Cardiomyocytes

Primary culture of cardiomyocytes was prepared from neonatal Sprague-Dawley rat. Briefly, rat heart was harvested and placed in ice-cold Ca^2+^- and bicarbonate-free Hanks' buffer. The ventricle was excised and minced. Minced ventricular tissues were dissociated by treatment with 0.09% trypsin 5 times at 37°C for 10 min. The supernatants of the first digestion were discarded, but other four supernatants were saved in Dulbecco's modified Eagle's medium (DMEM) containing 10% fetal bovine serum (FBS, Hyclone Corp., South America) and then centrifuged for 5 min at 900 ×g. Resuspended cells were then placed in a culture bottle at 37°C in a 5% CO_2_–95% O_2_ incubator for 1.5 h to adhere fibroblast. Then, nonadherent cells were counted with a hemocytometer, and the final myocyte cultures with over 90% cardiomyocytes were used for further experiments. One part of cardiomyocytes were cultured in 35 mm culture dishes at 37°C in a 5% CO_2_–95% O_2_ incubator, and the 3–5 d cultured cells were used for patch clamp recording. And other cardiomyocytes cultured in gelatin-coated plates incubated for 3–5 days were used for the experiment of A/R injury.

### 2.5. A/R Injury Model

The prepared cardiomyocytes were randomly divided into 9 groups: Sham anoxia group, A/R group, 1 mM nifedipine group, 1 *μ*M Y-27632 group, and TFR 3.7, 11.1, 33.3, 100, and 300 mg/L groups. Except that sham anoxia group was incubated under normoxic conditions, other groups were subjected to anoxia followed by reoxygenation. Anoxia was induced by changing the air content with 95% N_2_ and 5% CO_2_ gas mixture in a metabolic chamber for 3 h. Then the cultures were reoxygenated for 12 h by incubating the cells in 95% O_2_ and 5% CO_2_. Nifedipine, Y-27632, and TFR were, respectively, added into the DMEM at 24 h before anoxia.

Another set of experiment was performed for Western blot assay under the same conditions and grouping.

### 2.6. Measurement of MTT, LDH, MDA, and cTnT Levels

After A/R model, 20 *µ*L MTT solution (5 mg/mL) was added into each well and incubated for an additional 4 h. Then, 100 *μ*L DMSO was added into cardiomyocyte cultures to dissolve the formazan particulates. Finally, absorbance at 490 nm was measured using a microplate reader for the MTT assay.

Supernatants of cardiomyocyte cultures were collected; MDA content and LDH activity were, respectively, detected at 532 nm and 450 nm by spectrophotometry according to the procedures provided by the assay kits.

The level of cTnT in supernatants was measured by the method of immunoassay [15].

### 2.7. Western Blot Analysis

Cardiomyocytes were lysed by the solution containing 0.4 mmol L^−1^ phenylmethanesulfonyl fluoride (pH 7.4), separated by sodium dodecyl sulfate polyacrylamide gel electrophoresis on 10% polyacrylamide-Tris gels (Beyotime, China), and then transferred to a polyvinylidene difluoride membrane. Membranes were blocked by buffer (5% skim milk and 0.05% Tween 20 in Tris-buffered saline) at room temperature for 2 h and then incubated (4°C, overnight) with the same buffer containing rabbit polyclonal antibodies against ROCK1, or ROCK2 (Enogene, China) or monoclonal antibody against *β*-actin (Bioworld). After incubation with anti-rabbit second antibody (ZSGB Bio company, China, 1 : 10000 dilution in 5% skim milk) for 1 h at room temperature, the bands were visualized using an enhanced chemiluminescence kit (Thermo, USA). The intensity of immunoreactive bands was quantified with the use of an imaging densitometer.

### 2.8. Whole-Cell Patch Clamp Recording

As previously described [16], K^+^ currents in single rat cardiomyocyte were recorded with an EPC 10 patch clamp amplifier (HEKA Elektronik, Lambrecht/Pfalz, Germany) with Pulse and Pulse-fit software. 500 *μ*L of rat cardiomyocytes suspension was placed in a perfusion chamber on a microscope stage and superfused with Tyrode's solution (mmol·L^−1^: 143 NaCl, 5.4 KCl, 0.33 NaH_2_PO_4_, 1.8 CaCl_2_, 0.5 MgCl_2_, 5 HEPES, and 11 glucose, adjusted pH to 7.4 with NaOH). Micropipette patch pipette (resistance, 2.5~3 MΩ) was filled with intercellular fluid (mmol L^−1^: 135 KCl, 5 NaCl, 10 HEPES, 5 EGTA, 10 *μ*M 4,4′-diisothiocyano-2,2′-stilbenedisulfonic acid, and 5 Mg-ATP, adjusted pH to 7.2 with KOH). K^+^ current was normalized through dividing current by cell capacitance to get current density (pA/pF). Rat cardiomyocyte was held at −80 mV and voltage steps ranging from −130 to + 70 mV were applied for 500 ms in 20 mV increments. After rupture of the membrane, cardiomyocyte capacitance was estimated by a composite of the capacitance current and compensation of cell capacitor and pipette series resistance. The measurements can be made only if seals are in the gigaohm range. BaCl_2_ or TFR was dissolved in the extracellular solution and directly given to cardiomyocyte using injection port setting. Changes in K^+^ currents were observed in the same cell before and after administration of BaCl_2_ or TFR.

### 2.9. Statistical Analysis

Data are presented as means ± SD. Statistical analyses were performed with one-way ANOVA followed by the Duncan test to determine the differences between groups. A value of *P* < 0.05 was regarded as statistically significant.

## 3. Result

### 3.1. TFR Inhibits I/R-Induced Myocardial Infarction

**Figure 1:**
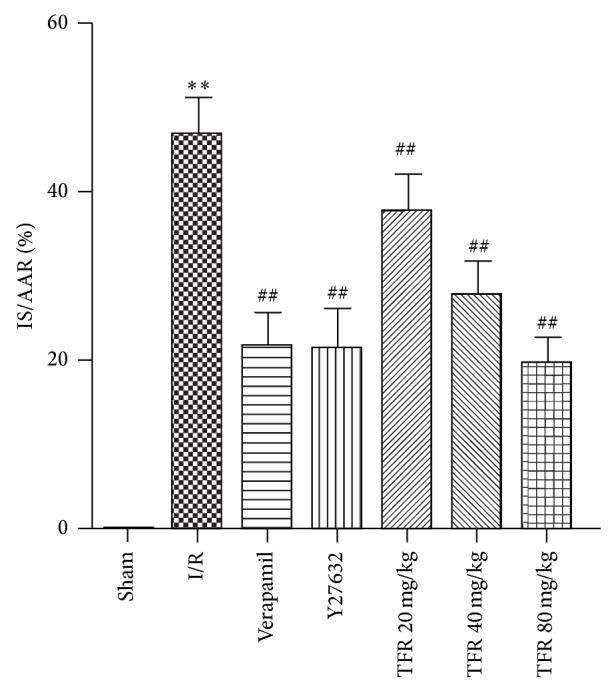
Effects of total flavones of* Rhododendron simsii* Planch flower (TFR), verapamil, and Y27632 on ischemia/reperfusion- (I/R-) induced myocardial infarction in rat. Infarct size was expressed as a percentage of the area at risk (IS/AAR). Results are means ± SD of 6 experiments. ^**^ *P* < 0.01 compared to sham; ^##^ *P* < 0.01 compared to I/R.

As shown in Figure 1, occlusion of LAD followed by reperfusion induced obvious myocardial injury as indicated by measurement of the IS/ARR ratio. The IS/AAR ratio was 46.3% ± 6.2% in I/R group (^**^ *P* < 0.01 versus sham group). 20, 40, and 80 mg/kg TFR significantly decreased the IS/ARR ratio, respectively (^##^ *P* < 0.01 versus sham group). Similarly, verapamil 1.6 mg/kg and Y27632 30 mg/kg also reduced the IS/ARR ratio (^##^ *P* < 0.01 versus sham group).

### 3.2. Effect of TFR on Viability of Rat Cardiomyocytes

**Figure 2:**
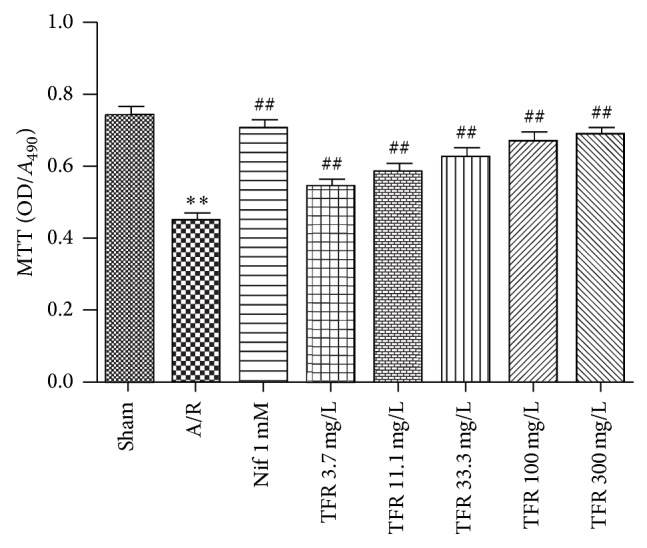
Effects of total flavones of* Rhododendron simsii* Planch flower (TFR) and nifedipine (Nif) on the viability of rat cardiomyocytes subjected to anoxia/reoxygenation (A/R) (MTT assay). Results are means ± SD of 5 experiments. ^*^ *P* < 0.01 compared to sham anoxia; ^##^ *P* < 0.01 compared to A/R.

Viability of rat cardiomyocytes was validated by MTT assay.  Figure 2 showed that A/R induced a significant reduction of viability of neonatal rat cardiomyocytes compared to that in sham anoxia group (^**^ *P* < 0.01 versus sham anoxia group). In the range of 3.7~300 mg/L, TFR markedly and concentration-dependently increased the viability of neonatal rat cardiomyocytes with an EC_50_ of 15.9 mg/L. Similar to TFR, 1 mmol/L nifedipine also increased the viability of rat cardiomyocytes (^##^ *P* < 0.01 versus A/R group).

### 3.3. Effect of TFR on LDH Activity and cTnT Level

**Figure 3:**
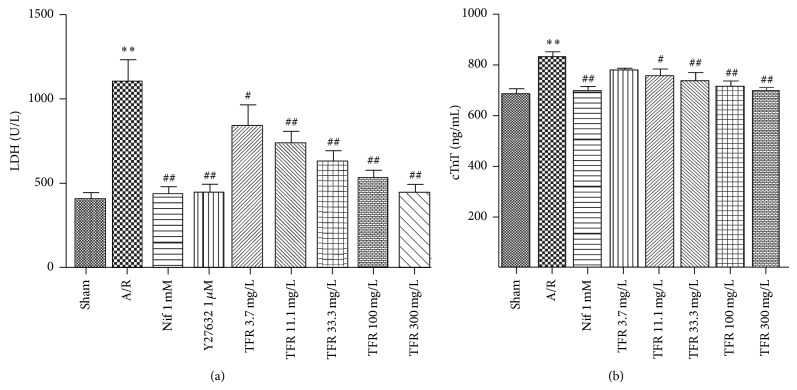
Effects of total flavones of* Rhododendron simsii* Planch flower (TFR), nifedipine (Nif), and Y27632 on lactate dehydrogenase (LDH) activity and cardiac troponin T (cTnT) level in the culture medium of neonatal rat cardiomyocytes subjected to anoxia/reoxygenation (A/R). (a) LDH activity; (b) cTnT level. Results are means ± SD of 5 experiments. ^**^ *P* < 0.01 compared to sham anoxia; ^#^ *P* < 0.05, ^##^ *P* < 0.01 compared to A/R.

Leakage of LDH or cTnT from cell into the culture medium is a major indicator of myocardial A/R injury. Significant increases of LDH activity and cTnT level in the culture medium of neonatal rat cardiomyocytes were detected in A/R group (^**^ *P* < 0.01 versus sham anoxia group). Treatment with TFR (3.7, 11.1, 33.3, 100 and 300 mg/L) or nifedipine 1 mmol/L markedly inhibited A/R-induced increases of LDH activity and cTnT level in culture medium (^#^ *P* < 0.05 or ^##^ *P* < 0.01 versus A/R group) (Figures 3(a) and 3(b)).

### 3.4. Effect of TFR on Production of MDA

**Figure 4:**
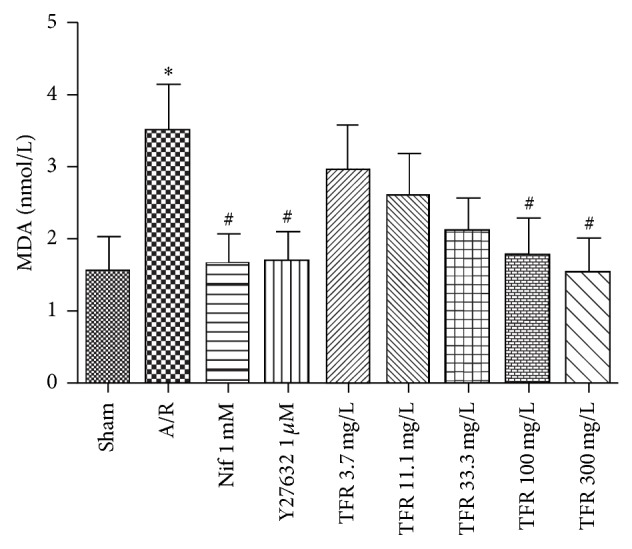
Effects of total flavones of* Rhododendron simsii* Planch flower (TFR) and nifedipine (Nif) on lactate dehydrogenase malondialdehyde (MDA) in the culture medium of neonatal rat cardiomyocytes subjected to anoxia/reoxygenation (A/R). Results are means ± SD of 5 experiments. ^*^ *P* < 0.05 compared to sham anoxia; ^#^ *P* < 0.05 compared to A/R.

There is a significant increase of MDA content in A/R group (^**^ *P* < 0.01 versus sham anoxia group). 3.7, 11.1, 33.3, 100, and 300 mg/L TFR obviously reduced the MDA content compared with A/R group. Treatment of nifedipine 1 mmol/L had a similar effect in reducing MDA production (^#^ *P* < 0.05 versus A/R group) (Figure 4).

### 3.5. Effect of Y27632 on A/R-Induced Injury of Rat Cardiomyocyte

As shown in Figures 3(a) and 4, treatment with Y27632 1 *μ*mol/L dramatically inhibited A/R-induced increases of LDH activity in culture medium (^##^ *P* < 0.01 versus A/R group). And Y27632 also obviously decreased the MDA content compared with A/R group (^##^ *P* < 0.01 versus A/R group).

### 3.6. Effect of TFR on ROCKs Protein Expression

**Figure 5:**
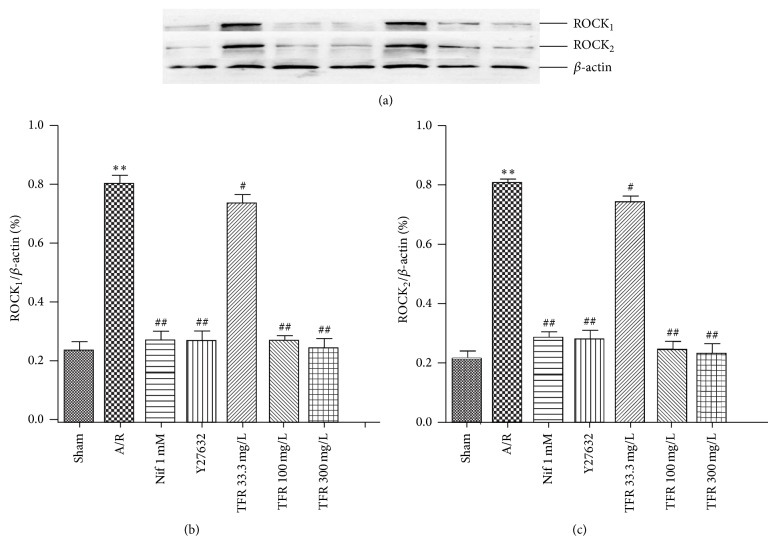
Effects of total flavones of* Rhododendron simsii* Planch flower (TFR) and Y23672 on expressions of ROCK_1_ and ROCK_2_ proteins in neonatal rat cardiomyocytes subjected to anoxia/reoxygenation (A/R). (a) Representative western blot analysis of expressions of ROCK_1_ and ROCK_2_. (b) Quantification of ROCK_1_ expression. (c) Quantification of ROCK_2_ expression. ROCK_1_, ROCK_2_, and *β*-actin were analyzed and quantified by densitometric analysis. Data are means ± SD of ROCK_1_/*β*-actin or ROCK_2_/*β*-actin from 3 experiments. ^**^ *P* < 0.01 compared to sham anoxia; ^#^ *P* < 0.05, ^##^ *P* < 0.01 compared to A/R.

The expressions of both ROCK_1_ and ROCK_2_ proteins were found in each group (Figure 5(a)), and levels of ROCK_1_ and ROCK_2_ proteins were quantified by using the densitometry (Figures 5(b) and 5(c)). Exposure to A/R significantly increased both ROCK_1_ and ROCK_2_ protein levels (^**^ *P* < 0.01 versus sham anoxia group). The increases of ROCK_1_ and ROCK_2_ were markedly inhibited by ROCK inhibitor Y27632 1 *μ*mol/L or TFR 33.3, 100, and 300 mg/L (^#^ *P* < 0.05 or ^##^ *P* < 0.01 versus A/R group). 1 mmol/L nifedipine had a similar effect on the expressions of ROCK_1_ and ROCK_2_.

### 3.7. K^+^ Currents in Neonatal Rat Ventricular Cardiomyocytes

**Figure 6:**
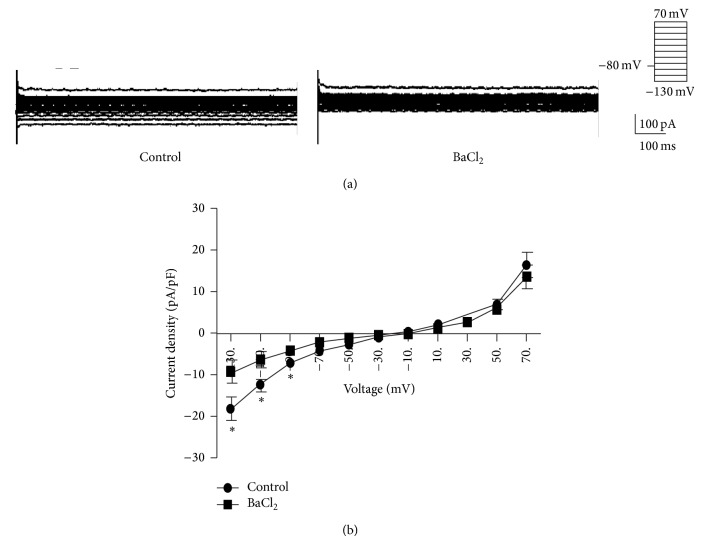
K^+^ currents in neonatal rat ventricular cardiomyocytes and effect of BaCl_2_ on the currents. (a) Traces of outward and inward K^+^ currents without or with 100 *μ*mol/L BaCl_2_. (b) Curves of current-voltage relationships of outward and inward K^+^ currents. Results are means ± SD of 5 experiments. ^*^ *P* < 0.05 compared to control.

To explore protective mechanisms of TFR on myocardial A/R injury, the effect of TFR on K^+^ channel in rat cardiomyocytes was investigated using the patch clamp method. Under current clamp mode, outward K^+^ current was evoked in rat cardiomyocytes from a holding of 10 or 30 mV to 70 mV, and inward K^+^ current was elicited from a holding of −30 or −50 mV to −130 mV (Figure 6(a)). Both outward and inward K^+^ currents were voltage-dependent. Exposure of cardiomyocytes to BaCl_2_ (100 *μ*M), a relatively selective inward rectifier K^+^ (Kir) channel inhibitor, obviously suppressed the inward K^+^ current (^*^ *P* < 0.05 versus control group) without effect on outward K^+^ current (Figures 6(a) and 6(b)). The results indicate inward K^+^ current evoked in rat cardiomyocytes was carried by Kir channel.

**Figure 7:**
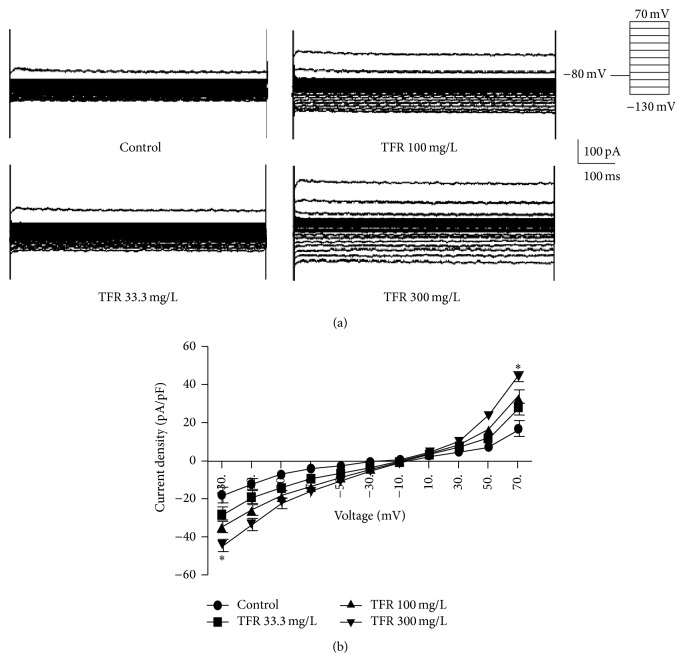
Effect of total flavones of* Rhododendron simsii* Planch flower (TFR) on K^+^ currents in neonatal rat ventricular cardiomyocytes. (a) Traces of outward and inward K^+^ currents without or with 33.3, 100, and 300 mg/L TFR. (b) Curve of current-voltage relationships of outward and inward K^+^ currents. Results are means ± SD of 5 experiments. ^*^ *P* < 0.05 compared to control.

Figure 7 displays that 300 mg/L TFR markedly augmented the inward current and outward K^+^ currents (^*^ *P* < 0.05 versus control group), suggesting that TFR could activate Kir channel and other types of K^+^ channels to cause K^+^ currents in rat cardiomyocytes.

## 4. Discussion

In the present study, we have found that (1) TFR has protective effects against myocardial I/R and A/R injury in rat; (2) ROCKs mediate protective effect of TFR on rat cardiomyocytes A/R injury; (3) TFR could promote opening of Kir channel and other types of K^+^ channels and increases K^+^ currents in rat cardiomyocytes.

The increase in infarct size is documented to be a reliable index of myocardial I/R injury. In this study, 30 min of ischemia followed by 90 min of reperfusion was noted to induce myocardial injury as assessed in terms of the increased IS/ARR ratio in rat heart. Like calcium channel blocker verapamil, TFR markedly reduced the IS/ARR ratio; the result indicates that TFR has a protective effect against myocardial I/R injury in rat.

LDH serves as an important metabolic enzyme in cardiomyocytes and could be leaked from injured cardiomyocytes. Hence, LDH level in the culture medium is a primary index to evaluate cell damage [17]. Troponin I is an inhibitory subunit of troponin that binds to actin in thin myofilaments to hold the actin-tropomyosin complex in place. There are three isoforms of troponin I: cardiac troponin I (cTnI), fast twitch skeletal muscle troponin I (fTnI), and slow twitch skeletal muscle troponin I (sTnI). cTnI is not present in serum from healthy people, but it can be detected in serum from patients with acute myocardium damage. Thus, cTnI in the culture medium is a sensitive and specific biochemical marker for detecting cardiomyocytes injury [9, 18–20]. In the present study, A/R-induced rat cardiomyocytes injury was detected as indicated by the decrease of cell viability and the increases of LDH and cTnT in culture medium, while treatment of TFR in the range of 3.7 to 300 mg/L significantly improved the aforementioned indexes including the increase of cell viability and reductions of LDH and cTnT in culture medium. Calcium antagonist nifedipine had comparable effects. These results indicate that TFR has a significant protective effect on A/R-injured rat cardiomyocytes.

A/R injury can produce large amounts of oxygen-free radicals in cardiomyocytes and subsequently causes lipid peroxidation and leads to cell damage. Thus, lipid peroxidation is one of mechanisms of cellular damage. MDA, a product of lipid peroxidation, has been applied to assess oxygen-free radicals-mediated myocardial I/R injury [21]. Our study revealed that 100 and 300 mg/L TFR significantly decreased the MDA level in culture medium; the result not only further indicated that TFR has protection on myocardial A/R injury, but also proposed that inhibition of lipid peroxidation may be, at least partially, involved in cardioprotective mechanism of TFR against myocardial A/R injury.

RhoA is one of the effecters of the small GTP-binding protein Rho. ROCK is ubiquitously expressed; it is the best-known downstream effector of RhoA. Both ROCK_1_ and ROCK_2_ are expressed in vascular smooth muscle and in myocardium [22–24], but their differential effects are still not well characterized. Increasing evidences have demonstrated that the RhoA-ROCK pathway plays a pivotal role in cardiovascular pathogenesis such as I/R injury, vascular smooth muscle cell (VSMC) proliferation, cardiac hypertrophy, heart failure, and ventricular remodeling [25, 26]. ROCK plays an important role in myocardial A/R damage, and inhibition of the RhoA-ROCK pathway has beneficial effects on both heart and vasculature functions. Treatment with the ROCK inhibitor Y-27632 or fasudil protected the heart against I/R injury and enhanced postischemia cardiac function [4, 24]. Our data indicate that ROCK inhibitor Y27632 markedly inhibited A/R injury-induced leakage of LDH and MDA production, demonstrating that Y27632 had protective effects on myocardial A/R injury. In vivo rat model of myocardial I/R, Y27632, also significantly reduced myocardial infarction.

Oxidative species activate the ROCK pathway [27–29]. RhoA expression is upregulated in ischemic myocardium and later activation of ROCKs occurs during reperfusion [4, 30]. Our results show that exposure to A/R injury significantly increased both ROCK_1_ and ROCK_2_ protein levels in rat cardiomyocytes, and the increases were markedly attenuated by treatment of 33.3, 100, and 300 mg/L TFR. Together with the facts that ROCK inhibitor Y27632 had similar suppression on A/R-induced increases of ROCK_1_ and ROCK_2_ expressions and protective effect on myocardial A/R injury, our results suggest that activation of ROCK during rat myocardial A/R injury and inhibition of ROCK were involved in the cardioprotection of TFR against myocardial A/R injury.

Accumulating studies indicate ROCK is a major regulator of the contractile proteins, including myosin light chain phosphatase (MLCP), myosin phosphatase target subunit, CPI-17, and myosin light chain (MLC). ROCK is responsible for VSMCs contraction in various vascular beds [23], and similar mechanism was made for cardiac contractility. ROCK inhibitor Y-27632 significantly suppressed the contraction in rabbit ventricular myocardium [31]. This suggests that the ROCK activation may at least partly contribute to cardiac contractility, and ROCK inhibition could decrease cardiac contractility. It is a known fact that the decrease of cardiac contractility could result in decreased cardiac work and reduction in myocardial oxygen demand. Thus, ROCK inhibitor-induced decrease of cardiac contractility may be also associated with its cardioprotection on I/R injury. Hyperin, one of the main effective components of TFR, was found to inhibit contraction of papillary muscles isolated from rabbit heart [32]; the study suggests that TFR might lead to a decrease of cardiac contractility through ROCK inhibition, which might aid in its cardioprotection.

K^+^ channel plays an important roles in diverse physiological processes. Multiple K^+^ channels such as voltage-dependent K^+^ (Kv) channel, Ca^2+^-activated K^+^ (K_Ca_) channel, K_ATP_ channel, and Kir channel have been identified in cardiomyocytes. RhoA-ROCK pathway also participates in modulation of K^+^ channel function. Activation of ROCK elicited Kv channel endocytosis and consequently attenuated Kv current in human embryonic kidney 293 cells, and this channel endocytosis was inhibited by ROCK inhibitor Y27632 [33]. It was noted that selective inhibition of ROCK could protect K_Ca_ channel function in rat cerebral arteries [28]. Thus, TFR might be at least having an indirect effect on the K^+^ channel via ROCK inhibition. In the present study, both inward K^+^ current and outward K^+^ current were elicited in neonatal rat cardiomyocytes. It is well-known that the inward K^+^ current is mediated by Kir channel, and our study shows that this inward K^+^ current was obviously suppressed by 100 *μ*mol/L BaCl_2_, a selective Kir channel inhibitor. The result indicates that this inward K^+^ current in neonatal rat cardiomyocytes is Kir current which is mediated by Kir channel. Kir current plays a key role in setting up the resting membrane potentials and the repolarization in cardiomyocytes [34]. Inhibition of Kir could abolish ischemic preconditioning-induced protection in rabbit ventricular cardiomyocytes [9]. Our data reveal that TFR significantly increased inward K^+^ current in rat cardiomyocytes, suggesting that TFR could promote the opening of Kir channel. Outward K^+^ current results in cell membrane hyperpolarization, which could cause the closure of voltage-dependent Ca^2+^ channel and subsequent reduction in Ca^2+^ influx. Our data indicate that TFR also markedly increased outward K^+^ current in rat cardiomyocytes; this implies that TFR could also open other types of K^+^ channels such as K_Ca_ channel or K_ATP_ channel. Therefore, the opening of K^+^ channels may partly contribute to cardioprotection of TFR on myocardial A/R injury in rat. However, outward K^+^ current is conducted by several types of K^+^ channels including K_Ca_ channel, Kv channel, K_ATP_ channel, and other K^+^ channels. It is necessary in future study to determine which types of K^+^ channels are responsible for TFR-increased outward K^+^ current and whether TFR activates K^+^ channels directly or indirectly via ROCK inhibition.

In summary, the present study shows that TFR has a significant protective effect on rat cardiomyocytes A/R injury through the improvement of cell viability, decreases of LDH, and cTnT releases and inhibition of MDA production. Our study is the first attempt to investigate the mechanisms of TFR against myocardial A/R injury. It was found that ROCKs inhibition and activation of K^+^ channels might mediate the cardioprotective effect of TFR.

## Abbreviations

TFR: Total flavones from* Rhododendron simsii* Planch flower
A/R: Anoxia and reoxygenation
I/R: Ischemia and reperfusion
ROCK: Rho-associated coiled-coil forming protein kinase
Kir: Inward rectifier potassium channel.
